# Cardiovascular Implications of Calcitonin Therapy: A Narrative Review of Mechanisms, Clinical Risks, and Safety Considerations

**DOI:** 10.7759/cureus.107708

**Published:** 2026-04-25

**Authors:** Jyothsna Goranti, Vaishnavi Sabesan, Sachin Sapkota, Vishak Prakash, Sandesh Murali

**Affiliations:** 1 Internal Medicine, Texas Tech University Health Sciences Center El Paso, El Paso, USA

**Keywords:** bisphosphonates, calcitonin, cardiovascular adverse effects, denosumab, drug safety, monitoring strategies, osteoporosis, pharmacovigilance

## Abstract

Calcitonin, a peptide hormone produced by thyroid parafollicular cells, is used clinically for acute hypercalcemia, Paget’s disease of bone, and, less commonly, postmenopausal osteoporosis. It also has recognized analgesic utility in select pain syndromes. Although its skeletal actions are well established, concerns remain regarding potential cardiovascular adverse effects, particularly in susceptible patients and with parenteral administration.

A narrative literature review was conducted using databases including PubMed and Google Scholar to identify publications evaluating the cardiovascular effects of calcitonin therapy. Clinical trials, observational studies, mechanistic investigations, case reports, and pharmacovigilance data were reviewed to summarize reported cardiovascular complications and potential underlying mechanisms.

Cardiovascular events associated with calcitonin therapy appear uncommon in clinical trials but have been reported in post-marketing surveillance and case reports. Documented manifestations include palpitations, arrhythmias, transient hypertension, chest discomfort, and peripheral edema. Proposed mechanisms include calcitonin-induced hypocalcemia, electrolyte disturbances such as hypokalemia, renal dysfunction, neuroendocrine activation, and altered vascular smooth muscle calcium signaling. Drug-drug interactions may further increase risk, particularly with medications sensitive to electrolyte shifts or those affecting cardiac conduction. Regulatory restrictions on certain formulations, especially intranasal calcitonin, reflect broader safety concerns, including potential malignancy signals.

Calcitonin remains clinically useful for selected indications, including acute hypercalcemia and refractory Paget’s disease. However, clinicians should be aware of potential cardiovascular risks, particularly in older adults and patients with renal impairment, electrolyte abnormalities, or concomitant arrhythmogenic medications. Baseline electrocardiography, electrolyte assessment, early follow-up monitoring, and patient education may be considered to mitigate potential risks. Further prospective studies and pharmacovigilance data are needed to better define the incidence and mechanisms of cardiovascular complications associated with calcitonin therapy.

## Introduction and background

Calcitonin is a 32-amino acid peptide hormone secreted by thyroid parafollicular (C) cells that contributes to calcium and phosphate homeostasis by inhibiting osteoclast-mediated bone resorption and increasing renal calcium excretion [1]. Therapeutically, calcitonin is available as human calcitonin and synthetic or animal-derived analogs, with salmon calcitonin most commonly used because of its higher receptor potency and longer duration of action compared with the human peptide [1]. It is administered primarily as an intranasal spray or by subcutaneous or intramuscular injection, with parenteral routes producing higher systemic exposure than intranasal therapy [1,2].

Historically, calcitonin was widely used for the treatment of postmenopausal osteoporosis, supported by early clinical trials demonstrating reductions in vertebral fracture risk [3,4]. However, its clinical role has declined because it produces smaller improvements in bone mineral density compared with bisphosphonates and because of safety concerns, including reports suggesting a possible increase in malignancy risk with prolonged intranasal use [4,5]. Consequently, most contemporary guidelines recommend reserving calcitonin for selected patients in whom first-line therapies are unsuitable or contraindicated [3].

Calcitonin remains clinically relevant as a second-line option for Paget’s disease of bone, particularly in patients who cannot tolerate bisphosphonates [6,7]. Clinical studies have demonstrated reductions in biochemical markers of bone turnover, although acquired resistance and reduced long-term efficacy have been reported in some patients [8,9]. Calcitonin is also used as a short-term adjunct in the management of acute hypercalcemia, where it provides rapid but transient reductions in serum calcium levels due to receptor downregulation and tachyphylaxis [1].

Beyond its approved indications, calcitonin has demonstrated analgesic properties and has been used in conditions such as vertebral compression fractures, neuropathic pain, and other chronic pain syndromes, likely through central neuromodulatory mechanisms in addition to antiresorptive effects [10-12].

Although the clinical use of calcitonin has declined with the availability of newer antiresorptive agents, it continues to be prescribed in specific clinical settings, including acute hypercalcemia, refractory Paget’s disease of bone, and selected cases of osteoporosis where first-line therapies are contraindicated [1,3,6]. These indications are encountered in diverse patient populations, particularly older adults and individuals with multiple comorbidities, many of whom already carry baseline cardiovascular risk [1].

Cardiovascular safety is biologically plausible because calcium plays a fundamental role in myocardial excitation-contraction coupling, sinoatrial and atrioventricular nodal conduction, vascular smooth muscle tone, and electrical repolarization [13-15]. Pharmacologic reductions in extracellular calcium may therefore alter myocardial excitability and predispose susceptible patients to arrhythmias or hemodynamic instability [16].

Despite its continued clinical use, the cardiovascular safety profile of calcitonin remains incompletely characterized. Existing evidence is scattered across clinical trials, pharmacovigilance reports, and case literature, and a comprehensive synthesis of the potential mechanisms and clinical relevance of cardiovascular adverse effects is lacking [16].

This narrative review summarizes reported cardiovascular adverse events associated with calcitonin therapy, outlines mechanistic hypotheses and clinically relevant drug interactions, and proposes monitoring strategies to reduce risk.

## Review

Literature search strategy

A narrative literature review was conducted using PubMed and Google Scholar to identify English-language publications evaluating the cardiovascular effects of calcitonin, including both human and salmon formulations. The search included literature available from database inception through January 2025. Search terms included calcitonin, cardiovascular, arrhythmia, QT prolongation, hypocalcemia, hypertension, edema, drug interaction, and pharmacovigilance databases. Eligible publications included clinical trials, observational studies, mechanistic investigations, case reports, and pharmacovigilance reports describing cardiovascular effects associated with calcitonin therapy. Editorials, opinion pieces without primary data, and studies not addressing cardiovascular outcomes were excluded. Clinical studies, mechanistic reports, and regulatory documents were reviewed to summarize reported adverse cardiovascular events and potential mechanisms. This narrative review was prepared in accordance with the principles outlined in the Scale for the Assessment of Narrative Review Articles (SANRA) to enhance transparency and methodological rigor.

Pharmacology and mechanism of action

Calcitonin exerts its physiological and pharmacologic effects through activation of the calcitonin receptor, a G protein-coupled receptor expressed on osteoclasts, renal tubular epithelial cells, and certain neurons [1,14]. Receptor activation stimulates intracellular signaling pathways, primarily cyclic adenosine monophosphate (cAMP) and the phospholipase C/inositol trisphosphate (PLC/IP₃) pathway [14].

In bone, calcitonin suppresses osteoclast-mediated bone resorption by reducing osteoclast motility, inhibiting differentiation of osteoclast precursors, and disrupting the acidic microenvironment required for matrix degradation [1,14]. These effects reduce calcium release from bone and contribute to its therapeutic role in conditions characterized by increased bone turnover.

In the kidney, calcitonin reduces tubular reabsorption of calcium and phosphate and promotes diuresis, thereby increasing urinary excretion of electrolytes and contributing to reductions in serum calcium concentrations [1]. 

The pharmacokinetic profile varies according to the route of administration. Intranasal formulations achieve peak plasma concentrations within approximately 15-40 minutes but demonstrate relatively low systemic bioavailability, whereas subcutaneous or intramuscular administration results in higher systemic exposure and peak levels within 15-30 minutes [1,2]. Calcitonin has a short plasma half-life ranging from minutes to several hours, undergoes renal metabolism, and is eliminated through both urine and feces [1].

Typical adult dosing includes 100 IU daily administered subcutaneously or intramuscularly, or 200 IU intranasally for osteoporosis; 100 IU daily for Paget’s disease; and 4 IU/kg every 12 hours for acute hypercalcemia, with dose escalation when clinically indicated [1].

Emerging formulations, including oral recombinant calcitonin, have been investigated in clinical trials to improve patient adherence, although these remain under evaluation and are not widely adopted in routine clinical practice [15].

Key therapeutic indications, pharmacokinetic characteristics, and clinically relevant adverse effects of calcitonin therapy are summarized in Table 1.

**Table 1:** Clinical indications, pharmacologic characteristics, and safety considerations of calcitonin therapy. Summary of major indications, dosing, mechanisms, pharmacokinetics, and clinically relevant adverse effects based on published literature.

Clinical Indication	Therapeutic Role	Typical Dosing and Administration	Mechanism of Action	Pharmacokinetic Profile	Adverse Events and Cardiovascular Considerations
Postmenopausal osteoporosis	Reserved for selected patients where first-line therapies (e.g., bisphosphonates) are unsuitable or contraindicated [1,3]	200 IU intranasally daily or 100 IU daily subcutaneously or intramuscularly [1]	Activates calcitonin receptors on osteoclasts, suppressing bone resorption and reducing calcium release from bone [1,14]	Low bioavailability via intranasal route (peak 15–40 min); higher systemic exposure via subcutaneous or intramuscular administration (peak 15–30 min); short half-life; renal metabolism [1,2]	Possible malignancy risk with prolonged intranasal use; potential cardiovascular risks, including arrhythmias or hemodynamic instability related to electrolyte shifts.
Paget’s disease of bone	Second-line option, particularly for patients intolerant to bisphosphonates [1,6]	100 IU daily subcutaneously or intramuscularly [1]	Inhibits osteoclast-mediated bone resorption and suppresses biochemical markers of bone turnover [1,14]	Peak levels reached within 15–30 minutes after parenteral administration; short half-life; eliminated through urine and feces [1]	Acquired resistance and reduced long-term efficacy (tachyphylaxis); possible cardiovascular instability related to electrolyte disturbances [8]
Acute hypercalcemia	Short-term adjunct for rapid but transient reduction in serum calcium levels [1]	4 IU/kg every 12 hours subcutaneously or intramuscularly; dose escalation may be required [1]	Increases renal calcium excretion and inhibits bone resorption, producing a rapid reduction in serum calcium [1]	Rapid peak following injection; rapid metabolism and short half-life; transient effects due to receptor downregulation [1,6]	Tachyphylaxis; potential myocardial excitability changes and arrhythmias related to reductions in extracellular calcium [1,16]
Vertebral compression fractures and neuropathic pain	Analgesic or adjunctive therapy for pain management [10–12]	Dosing varies by indication and route; intranasal or parenteral formulations are used in clinical practice [1]	Central neuromodulatory mechanisms in addition to antiresorptive effects [11,13]	Dependent on route of administration, short plasma half-life; renal metabolism [1]	General risks include the need for cardiovascular monitoring in susceptible patients [1,16]

Cardiovascular physiology and calcium homeostasis

Calcium plays a central role in myocardial excitation-contraction coupling, impulse conduction, and vascular smooth muscle tone [16]. Alterations in extracellular calcium concentrations can affect myocardial depolarization, prolong repolarization, and modify vascular resistance. Because calcitonin lowers serum calcium levels through both skeletal and renal mechanisms, it may influence cardiovascular physiology, particularly in susceptible patients.

Hypocalcemia may prolong the QT interval, increase myocardial excitability, and predispose to arrhythmias, especially when accompanied by electrolyte abnormalities such as hypokalemia or hypomagnesemia [16]. Hemodynamic instability may also occur in patients with impaired cardiac reserve or autonomic dysfunction.

Patients at higher risk of clinically significant effects include those with chronic kidney disease, structural heart disease, electrolyte disturbances, and polypharmacy, particularly individuals receiving medications that affect conduction or repolarization [1,16].

Cardiovascular adverse effects

Arrhythmias

Arrhythmias have been proposed as a potential cardiovascular complication of calcitonin therapy, although direct clinical evidence remains limited. Existing reports are primarily derived from mechanistic studies and isolated case reports rather than large clinical trials or cohort studies [1,16,17]. Proposed mechanisms include hypocalcemia-induced electrical instability and electrolyte disturbances [1,16]. Case reports have described severe arrhythmias occurring after parenteral calcitonin administration, sometimes in the setting of hypersensitivity reactions and transient electrolyte abnormalities [17].

Key risk factors associated with calcitonin-related arrhythmias and their proposed mechanisms are summarized in Table 2.

**Table 2:** Risk factors associated with arrhythmias and cardiovascular instability during calcitonin therapy. This table summarizes major patient-related and treatment-related factors that may increase the likelihood of arrhythmias or hemodynamic instability based on published literature.

Risk Factor	Biological Mechanism	Clinical Manifestation	Associated Conditions or Triggers
Pre-existing cardiovascular disease	Lowered arrhythmic threshold due to structural or electrical cardiac abnormalities [16]	Increased susceptibility to arrhythmias	Chronic cardiac comorbidities
Systemic hypersensitivity reactions	Histamine release and inflammatory mediator surge increase arrhythmogenic potential [17]	Allergic-mediated dysrhythmias	Anaphylaxis or systemic allergic reactions
Hypokalemia or other electrolyte abnormalities	Disruption of myocardial repolarization and triggered activity [16]	QT prolongation and ventricular arrhythmias	Electrolyte imbalances, diuretic use, renal disease
Concomitant medications	Pharmacodynamic or pharmacokinetic interactions affecting cardiac conduction [1,16]	Potentiation of arrhythmias	Digoxin, macrolides, antipsychotics and QT-prolonging drugs
Hypocalcemia	Reduced extracellular calcium leading to increased myocardial excitability and prolonged repolarization [1,16]	Increased myocardial excitability and arrhythmias	Rapid calcium shifts, renal dysfunction
Rapid parenteral administration	Acute shifts in myocardial calcium handling and rapid electrolyte changes [1]	Acute-onset arrhythmias	Intravenous or intramuscular administration

Hypertension and Chest Pain: Episodes of hypertension and chest discomfort have occasionally been described in association with calcitonin therapy, although direct clinical evidence remains limited and causality has not been firmly established. Most available literature discusses physiologic mechanisms involving calcium-regulating hormones and neuroendocrine responses rather than well-documented adverse-event series. Neuroendocrine activation has also been observed, with increases in ACTH, beta-endorphin, and cortisol levels following calcitonin administration [13].

These hormonal changes may enhance vascular reactivity and catecholamine sensitivity, potentially contributing to transient elevations in blood pressure or vasospastic symptoms. Although these effects are typically transient, patients with pre-existing hypertension or autonomic instability may be more susceptible.

The mechanisms underlying chest pain in this context are likely multifactorial and may include vasospasm, transient hemodynamic changes, or electrolyte disturbances rather than fixed coronary obstruction. Overall, the available data are largely mechanistic or observational and should therefore be interpreted cautiously, with these associations considered hypothesis-generating rather than definitive evidence of a causal relationship.

Drug-Drug Interactions and Rate-Limiting Agents

Potential drug interactions with calcitonin have been proposed, particularly with medications that are renally cleared or that influence myocardial conduction. Direct clinical evidence documenting these interactions is limited; most proposed interactions are based on pharmacodynamic considerations such as electrolyte-mediated effects on myocardial excitability and drug sensitivity [1].

Caution is also advisable when calcitonin is administered with calcium channel blockers or diuretics, as additive effects on electrolyte balance, conduction, or hemodynamics may occur. Concomitant use with bisphosphonates may increase the risk of hypocalcemia due to overlapping mechanisms of reduced bone resorption [7]. 

 A summary of clinically relevant interactions is presented in Table 3.

**Table 3:** Drug classes with potential interactions or additive cardiovascular risks during calcitonin therapy. This table summarizes pharmacodynamic or mechanistically plausible interactions that may increase the risk of electrolyte disturbances, arrhythmias, or hemodynamic changes. Many of these interactions are theoretical and based on physiologic considerations rather than direct clinical studies.

Drug Class	Example Agents	Mechanism and Impact	Clinical Implication
Cardiac glycosides	Digoxin	Calcitonin-induced reductions in serum calcium may increase myocardial sensitivity to digoxin [1,16]	Increased risk of digoxin toxicity, including arrhythmias and gastrointestinal symptoms
Loop diuretics	Furosemide	Synergistic electrolyte disturbances, including hypocalcemia or hypokalemia [1,16]	Increased risk of electrolyte imbalance and arrhythmias
Bisphosphonates	Alendronate, zoledronic acid	Additive hypocalcemic effects due to overlapping mechanisms of reduced bone resorption [1,7]	Increased risk of symptomatic hypocalcemia
Calcium channel blockers	Verapamil, diltiazem	Potential alterations in renal clearance or additive effects on conduction; clinical evidence is limited [1,18,19]	Possible increased risk of bradycardia or hypotension; monitoring advised

Edema

Peripheral or generalized edema has been reported in association with calcitonin therapy, although its incidence is not well defined [20,21]. The mechanism is not fully understood but may involve alterations in vascular smooth muscle calcium signaling, changes in sodium and water handling, and effects on vascular permeability. Patients with heart failure, chronic kidney disease, or baseline volume overload may be particularly vulnerable, and monitoring for fluid retention is advisable during therapy.

Indirect Cardiovascular Effects

Gastrointestinal symptoms such as nausea, vomiting, and diarrhea may lead to dehydration and electrolyte disturbances that increase arrhythmia risk [1]. Rare complications such as pancreatitis have also been reported [22]. Metabolic complications, particularly hypocalcemia, may cause neuromuscular symptoms and cardiac rhythm disturbances [23,24].

Hypersensitivity reactions, including bronchospasm and anaphylaxis, have been reported and may result in acute hemodynamic instability requiring urgent treatment, including standard anaphylaxis management measures when indicated [1,17]. Neuropsychiatric symptoms such as depression and dizziness, genitourinary effects including polyuria and nocturia, and systemic adverse effects have also been described [24]. Intranasalformulations may also produce local nasal adverse effects, including mucosal irritation, crusting, and epistaxis [25]. Long-term exposure to intranasal calcitonin has been associated with a modest increase in malignancy risk in some analyses, leading to regulatory restrictions in certain indications [26,27].

Calcitonin therapy is associated with a range of systemic and local adverse effects, some of which may indirectly contribute to cardiovascular instability through electrolyte disturbances, hypersensitivity reactions, or hemodynamic changes (Table 4).

**Table 4:** Adverse effects and systemic complications associated with calcitonin therapy.

Adverse Effect Category	Specific Symptom or Condition	Formulation or Route	Clinical Impact	Relative Clinical Concern
Oncology	Malignancy risk	Intranasal	Regulatory restrictions in certain indications due to increased cancer risk reported in long-term studies [26,27]	Moderate
Immune system	Hypersensitivity reactions (bronchospasm, anaphylaxis)	General	Acute hemodynamic instability requiring urgent treatment; rare but potentially severe reactions reported in case literature and pharmacovigilance data [1,17]	High
Gastrointestinal (Inflammatory)	Pancreatitis	General	Rare inflammatory complication reported in case literature; causality uncertain but requires clinical vigilance [22]	High
Metabolic	Hypocalcemia	General	May cause neuromuscular symptoms, QT prolongation, and cardiac rhythm disturbances [1,24]	Moderate
Gastrointestinal (Common adverse effects)	Nausea, vomiting, diarrhea	General	May lead to dehydration and electrolyte disturbances increasing arrhythmia risk [1]	Moderate
Respiratory / Local nasal	Mucosal irritation, crusting, epistaxis	Intranasal	Local irritation and nasal bleeding commonly reported with intranasal formulations [25]	Low
Neuropsychiatric	Depression and dizziness	General	Dizziness and central effects reported in pharmacovigilance data; usually mild [1,24]	Low
Genitourinary	Polyuria and nocturia	General	Increased urinary calcium excretion may contribute to diuresis and volume shifts [1,24]	Low

Clinical case reports and real-world data

Published evidence regarding cardiovascular adverse events associated with calcitonin therapy includes isolated case reports and signals from pharmacovigilance reporting systems. However, systematic analyses quantifying the incidence of such events are limited. Spontaneous reporting systems such as the FDA Adverse Event Reporting System (FAERS) and EudraVigilance primarily serve to identify potential safety signals rather than establish causal relationships or incidence estimates. Consequently, these observations should be interpreted cautiously and considered hypothesis-generating.

Risk stratification, monitoring, and mitigation

Before initiating calcitonin therapy, baseline evaluation should include electrocardiography, serum calcium, potassium, magnesium, and renal function testing, particularly in patients with cardiovascular comorbidities or polypharmacy [28]. Follow-up monitoring is advisable within one to two weeks after initiation or dose adjustment, and earlier if symptoms such as palpitations, dizziness, or chest discomfort develop.

Preventive strategies include calcium and vitamin D supplementation when appropriate, careful review of interacting medications, and patient education regarding warning symptoms requiring prompt medical evaluation [1,29].

Regulatory status and post-marketing surveillance

Concerns regarding long-term safety, including malignancy signals and limited comparative efficacy, have led regulatory agencies to restrict the use of calcitonin, particularly intranasal formulations for osteoporosis. Injectable formulations remain available but are generally recommended for short-term or selected indications. Ongoing pharmacovigilance continues to inform prescribing practices and safety recommendations.

Future directions

Future research should focus on prospective safety registries, mechanistic studies examining calcitonin’s effects on cardiac ion channels and myocardial excitability, and pharmacogenomic approaches to identify individuals at increased risk of adverse events. Development of receptor-selective analogs with improved safety profiles may allow more targeted therapeutic applications while minimizing cardiovascular risk.

Limitations

Because this article was designed as a narrative review synthesizing evidence from heterogeneous sources, including clinical trials, observational studies, case reports, and pharmacovigilance data, a formal risk-of-bias or evidence grading framework was not applied. Consequently, findings from different study designs should be interpreted cautiously, as lower-level evidence, such as case reports, primarily provides hypothesis-generating observations rather than definitive causal inference. Additional limitations include the restricted database search strategy and the absence of a formal systematic review methodology, which may introduce selective citation bias. Furthermore, many of the cardiovascular associations discussed are derived from mechanistic reasoning or isolated reports, and robust epidemiologic data quantifying the incidence of these events remain limited.

## Conclusions

Calcitonin continues to have a limited but clinically relevant role in selected situations, particularly in the short-term management of acute hypercalcemia and as an alternative therapy for Paget’s disease of bone in patients who cannot tolerate first-line agents. Although its use in osteoporosis has declined with the availability of more effective antiresorptive therapies, calcitonin remains part of the therapeutic armamentarium in specific clinical contexts.

The available literature suggests that cardiovascular events such as arrhythmias, transient hypertension, chest discomfort, and fluid retention may occur during calcitonin therapy; however, the overall evidence base remains limited and heterogeneous. Much of the existing data is derived from mechanistic studies, case reports, and pharmacovigilance observations rather than large controlled clinical studies. Consequently, these associations should be interpreted cautiously and viewed primarily as hypothesis-generating signals rather than definitive causal relationships.

In clinical practice, awareness of potential cardiovascular effects and consideration of patient-specific risk factors, including electrolyte disturbances, renal dysfunction, and underlying cardiovascular disease, may help guide prudent use of calcitonin therapy. Such considerations are largely precautionary and based on physiologic reasoning rather than direct evidence from prospective studies.

Future research should focus on systematic pharmacovigilance analyses, prospective safety registries, and mechanistic studies evaluating calcitonin’s effects on myocardial electrophysiology and vascular physiology. Improved characterization of these potential risks will help clarify the clinical significance of reported cardiovascular events and support more evidence-informed prescribing practices.
